# Das Reidentifikationspotenzial von strukturierten Gesundheitsdaten

**DOI:** 10.1007/s00103-023-03820-2

**Published:** 2024-01-17

**Authors:** Jörg Drechsler, Hannah Pauly

**Affiliations:** 1 Institut für Arbeitsmarkt- und Berufsforschung (IAB), Regensburger Str. 104, 90478 Nürnberg, Deutschland; 2 Universität Mannheim, Mannheim, Deutschland; 3 Joint Program in Survey Methodology (JPSM), University of Maryland, College Park, MD USA; 4 Forschungsdatenzentrum Gesundheit, Bundesinstitut für Arzneimittel und Medizinprodukte (BfArM), Bonn, Deutschland

**Keywords:** Reidentifikationsrisiko, Anonymisierung, Synthetische Daten, Elektronische Patientenakte, Datenschutz, Reidentification risk, Anonymization, Synthetic data, Electronic health records, Data privacy

## Abstract

Ein breiter Zugang zu Gesundheitsdaten bietet enormes Potenzial für Wissenschaft und Forschung. Allerdings enthalten Gesundheitsdaten oftmals sensible Informationen, die es in besonderer Weise zu schützen gilt. Vor diesem Hintergrund befasst sich dieser Artikel mit dem Reidentifikationspotenzial von Gesundheitsdaten. Nach einer Abgrenzung der Begrifflichkeiten werden Faktoren diskutiert, die das Reidentifikationspotenzial beeinflussen. Es wird Bezug auf internationale Standards zum Schutz von Gesundheitsdaten genommen und die Wichtigkeit von verfügbarem Hintergrundwissen erläutert. Auf Basis des Zwischenfazits, dass das Reidentifikationspotenzial häufig unterschätzt wird, werden verschiedene Möglichkeiten zu dessen Reduzierung anhand des Konzepts der *Five Safes* vorgestellt. In diesem Zusammenhang wird sowohl auf klassische Anonymisierungsverfahren als auch auf Möglichkeiten zur Generierung synthetischer Gesundheitsdaten eingegangen. Der Beitrag schließt mit einem Fazit und kurzem Ausblick auf das kommende Forschungsdatenzentrum Gesundheit beim Bundesinstitut für Arzneimittel und Medizinprodukte.

## Abstract (en)

Broad access to health data offers great potential for science and research. However, health data often contains sensitive information that must be protected in a special way. In this context, the article deals with the re-identification potential of health data. After defining the relevant terms, we discuss factors that influence the re-identification potential. We summarize international privacy standards for health data and highlight the importance of background knowledge. Given that the reidentification potential is often underestimated in practice, we present strategies for mitigation based on the *Five Safes* concept. We also discuss classical data protection strategies as well as methods for generating synthetic health data. The article concludes with a brief discussion and outlook on the planned Health Data Lab at the Federal Institute for Drugs and Medical Devices.

## Einleitung

In unserer heutigen Gesellschaft gewinnen Daten eine immer größere Bedeutung und prägen zunehmend alle Aspekte unseres täglichen Lebens. Egal ob zur Optimierung der Geschäftsprozesse in der Wirtschaft, für die evidenzbasierte Politikberatung oder für die aktuelle Forschung, Daten bilden das Rückgrat des gesellschaftlichen Fortschritts. Dies gilt insbesondere auch für die Medizin.

Auswertungen von Medizindaten können zu einer Verbesserung der Gesundheitsversorgung beitragen. So können beispielsweise Abrechnungsdaten von Krankenversicherungen Aufschlüsse über den Zusammenhang zwischen gestellten Diagnosen und durchgeführten Therapien liefern. Umgekehrt werden im medizinischen Bereich die Auswirkungen mangelnder Verfügbarkeit von Daten besonders deutlich. Während der Coronakrise wurde die mangelnde Datenlage wiederholt kritisiert.^1^^,^^2^ Eine umfangreiche Datenbasis und ein möglichst breiter Zugang zu diesen Daten sind daher von hohem gesamtgesellschaftlichen Interesse.

Allerdings darf trotz der enormen Relevanz einer umfänglichen Datenverfügbarkeit der Schutz der Privatsphäre nicht außer Acht gelassen werden. Datenskandale wie der Verkauf von Facebook-Daten an Cambridge Analytica zeigen, dass in Zeiten, in denen große Datenbestände eine lukrative Einnahmequelle bieten, der Schutz der Privatsphäre Gefahr läuft, ins Hintertreffen zu geraten. Zudem führt die ubiquitäre Verfügbarkeit von Daten zu einem stetig wachsenden Reidentifikationsrisiko. Frei verfügbare Datenquellen können zunehmend dazu genutzt werden, Personen in vermeintlich anonymisierten Datensätzen zu reidentifizieren (z. B. [1, 2]). Ein weiterer Faktor, der das Reidentifikationsrisiko in den letzten Jahren deutlich erhöht hat, ist die stark gestiegene Rechenleistung, die *Reconstruction Attacks*, wie den simulierten Angriff auf die Zensusdaten des U.S. Census Bureaus [3], überhaupt erst möglich gemacht haben.

In der Praxis ist daher eine sorgfältige Abwägung zwischen größtmöglichem Erkenntnisgewinn durch breiten Datenzugang und dem Schutz der Privatsphäre der Beobachtungseinheiten (neben natürlichen Personen können beispielsweise auch Krankenhäuser oder andere Leistungserbringer betroffen sein) immer wieder von Neuem erforderlich. Insbesondere bei sensiblen Informationen wie Gesundheitsdaten muss stets sichergestellt werden, dass der Datenschutz gewahrt bleibt. Neben moralischen und ethischen Abwägungen spielt die geltende Rechtsaufassung bei der Frage der Datenweitergabe eine zentrale Rolle. So findet beispielsweise die Datenschutzgrundverordnung (DSGVO) nur dann Anwendung, wenn es sich um personenbezogene Daten handelt. Allerdings wird in Artikel 4 der DSGVO klargestellt, dass der Begriff des Personenbezugs sehr weit zu fassen ist. Es reicht beispielsweise nicht, lediglich direkte Identifikatoren wie Namen und Adressen zu entfernen bzw. diese durch ein Kennzeichen zu ersetzen (Pseudonymisierung). Vielmehr heißt es im Erwägungsgrund 26 der DSGVO: „Um festzustellen, ob eine natürliche Person identifizierbar ist, sollten alle Mittel berücksichtigt werden, die von dem Verantwortlichen oder einer anderen Person nach allgemeinem Ermessen wahrscheinlich genutzt werden, um die natürliche Person direkt oder indirekt zu identifizieren, wie beispielsweise das Aussondern.“ Es geht also darum, dass Reidentifikationsrisiko zu beurteilen. Nur wenn dieses Risiko vernachlässigbar erscheint, kann davon ausgegangen werden, dass die DSGVO nicht greift.

Vor diesem Hintergrund befasst sich dieser Artikel mit dem Reidentifikationspotenzial bei Gesundheitsdaten. Insbesondere soll den Fragen nachgegangen werden, welche Faktoren das Reidentifikationspotenzial beeinflussen und wie dieses mitigiert werden kann. Nach einer allgemeinen Einführung in das Thema werden explizit die Risiken im Kontext von Gesundheitsdaten erörtert. Anschließend werden verschiedene Maßnahmen und Verfahren diskutiert, um das Reidentifikationsrisiko zu minimieren. Der Artikel schließt mit einem Fazit und Ausblick.

## Das Reidentifikationspotenzial

Die grundsätzliche Möglichkeit, eine einzelne Beobachtung in den Daten eindeutig zu identifizieren, wird als „Reidentifikationspotenzial“ bezeichnet. Enthält der Datensatz direkte Identifikatoren wie Name und Anschrift, ist eine Identifikation in den meisten Fällen unmittelbar möglich; das Reidentifikationspotenzial ist entsprechend hoch. Allerdings reicht das Entfernen der direkten Identifikatoren in vielen Fällen nicht aus, um das Reidentifikationspotenzial auf ein akzeptables Maß zu senken. Wie bereits in der Einleitung erwähnt, können externe Datenquellen mit direkten Identifikatoren dazu verwendet werden, einzelne Beobachtungen in dem pseudonymisierten Datensatz zu identifizieren. In der Fachliteratur werden die Merkmale, die auch in anderen Datenquellen verfügbar sind und damit zu Reidentifikationszwecken verwendet werden können, als Schlüsselvariablen bezeichnet. Nach einer erfolgreichen Identifikation auf Basis der Schlüsselvariablen können potenzielle Angreiferinnen und Angreifer sensible Informationen über die betroffenen Beobachtungen erhalten. Pauschal lässt sich sagen, dass mit einer steigenden Zahl an Schlüsselvariablen und detaillierterem Informationsgehalt (beispielsweise exakte Altersangaben statt Angaben in 5‑Jahres-Intervallen) das Reidentifikationspotenzial steigt.

### Reidentifikationspotenzial vs. Reidentifikationsrisiko

Während das Reidentifikationspotenzial lediglich beurteilt, ob und wie einfach eine Reidentifikation einzelner Beobachtungen möglich ist, berücksichtigt das Reidentifikationsrisiko zusätzlich, wie wahrscheinlich es ist, dass eine missbräuchliche Nutzung der Daten zur Identifikation einzelner Personen auch tatsächlich durchgeführt wird. Dieses Risiko hängt von zahlreichen Faktoren ab: Wer hat Zugriff auf die Daten? Wie vertrauenswürdig sind die Nutzenden? Welchen Nutzen bietet eine mögliche Reidentifikation für die Angreifenden? Wie leicht ist eine Reidentifikation durchführbar? Wie hoch ist die Wahrscheinlichkeit, dass der Angriff entdeckt wird?

All diese Faktoren müssen bei einer Nutzen-Risiko-Abwägung bezüglich der Bereitstellung der Daten berücksichtigt werden. So macht es beispielsweise einen großen Unterschied, ob die Daten nur in den Räumen der bereitstellenden Institution analysiert werden oder ob sie allgemein zugänglich auf einer Webseite zum Download angeboten werden. Diese Risikobeurteilung ist Kernbestandteil des *Five-Safes*-Konzepts [4], das in diesem Artikel noch ausführlicher erläutert wird.

## Reidentifikationspotenzial von Gesundheitsdaten

Der folgende Abschnitt gibt einen Überblick über Faktoren, die das Reidentifikationspotenzial von Gesundheitsdaten und Krankenkassenabrechnungsdaten im Speziellen beeinflussen können. Krankenkassenabrechnungsdaten sind strukturierte medizinische Informationen, die unter anderem die folgenden Themenbereiche umfassen können [5]: Versichertenstammdaten und Informationen über den Versicherungsverlauf, Informationen über Sterblichkeit, ambulante und stationäre ärztliche Versorgung (dabei insbesondere Diagnosen, durchgeführte Prozeduren oder Arzneimittelverordnungen), Informationen über Heilmittel, Disease-Management-Programme, Informationen über die zahnärztliche Versorgung oder Arbeitsunfähigkeit. Abrechnungsdaten können in verschiedenen Kontexten für die Sekundärdatennutzung zugänglich gemacht werden.

Zum Reidentifikationspotenzial von Krankenkassenabrechnungsdaten in Deutschland gibt es nur wenige der Autorin und dem Autor bekannte Untersuchungen. Daher umfasst die folgende Übersicht hauptsächlich internationale Literatur zum Reidentifikationspotenzial von strukturierten Gesundheitsdaten bzw. Abrechnungsdaten. Nicht betrachtet werden Studien zu unstrukturierten Daten wie Bild- oder Textdaten, sowie Studien, die ausschließlich ethische oder rechtliche Aspekte diskutieren oder ausschließlich eine Methode zur Deidentifizierung von Daten und deren Güte beschreiben. Weiterhin wird nur das Reidentifikationspotenzial, das für Patientinnen und Patienten bzw. Versicherte besteht, betrachtet. In der Praxis sind jedoch auch mögliche Reidentifikationsrisiken für Leistungserbringer, wie z. B. Ärztinnen und Ärzte oder Krankenhäuser, und Leistungsträger, wie Kranken- oder Unfallkassen, zu beachten.

### Einflussfaktoren für das Reidentifikationspotenzial

In Deutschland waren im Jahr 2020 ca. 73,36 Mio. Personen gesetzlich und ca. 8,73 Mio. Personen privat krankenversichert.^3^ Damit stehen Krankenkassenabrechnungsdaten über nahezu die gesamte Population in Deutschland zur Verfügung. Gemeinsam mit der Vielfalt der erhobenen Merkmale ergeben sich hieraus schnell Merkmalskombinationen, die in der Population einzigartig oder zumindest sehr selten sind. Dass einzigartige Merkmalskombinationen ein hohes Reidentifikationspotenzial haben, wurde in [1] gezeigt, indem Krankenversichertendaten mit einem Wählerverzeichnis anhand von Postleitzahl, Geburtsdatum und Geschlecht verknüpft wurden. Bei elektronischen Gesundheitsdaten bestehen häufig einzigartige Kombinationen demografischer Attribute. Eine weitere Studie [6] konnte zeigen, dass geschätzte 87 % der US-Bevölkerung für Geschlecht, Geburtsdatum und 5‑stellige Postleitzahl eine einzigartige Merkmalskombination aufweisen. Einzigartige Kombinationen können jedoch auch in Bezug auf Diagnosen, verordnete Arzneimittel oder durchgeführte Prozeduren bestehen. In [7] wurde herausgefunden, dass Datensätze allein auf Basis von Diagnosecodes verknüpft werden können, und in [8] wurde gezeigt, dass 96 % der betrachteten Patientinnen und Patienten eines Krankenhauses in Bezug auf die Kombination der Diagnosecodes in ihren elektronischen Patientenakten einzigartig sind.

Krankenkassenabrechnungsdaten werden über einen langen Zeitraum hinweg erhoben und gespeichert. Damit stehen für einzelne Personen longitudinale Daten über mehrere Jahre hinweg zur Verfügung, was das Reidentifikationspotenzial erhöhen kann. Beispielsweise wurde gezeigt, dass der Anteil von Patientinnen und Patienten mit einzigartigen Kombinationen durchgeführter Labortests ansteigt, je häufiger diese Patientinnen und Patienten im Krankenhaus behandelt wurden [9]. Je mehr Einträge über eine Person vorhanden sind, desto höher ist die Wahrscheinlichkeit für einzigartige Merkmalskombinationen (s. auch [8]). In Krankenkassenabrechnungsdaten sind z. B. für alle Versicherten in der Regel jeweils mehrere Einträge zu ambulanten oder stationären Behandlungen vorhanden. Damit gibt es für alle Versicherten auch mehrere Einträge zu Diagnosen, verordneten Arzneimitteln etc. Einzelne dieser Merkmale können sehr stabil über die Zeit sein. Dies sind z. B. das Geburtsjahr, das Sterbedatum oder bei chronischen Erkrankungen Diagnosecodes oder verordnete Arzneimittel. In Attributen, die stabil über die Zeit bzw. replizierbar sind, kann ein höheres Reidentifikationspotenzial gesehen werden [10].

Nicht nur Kombinationen von Merkmalen, sondern auch die Ausprägungen einzelner Merkmale können ein hohes Reidentifikationspotenzial mit sich bringen. In einer Studie [11] wurden die statistische Häufigkeit und die phänotypische Erkennbarkeit von Merkmalsausprägungen bei medizinischen Routinedaten aus hausärztlichen Praxisinformationssystemen in Deutschland in die Bewertung des Reidentifikationspotenzials einbezogen. Zusätzlich haben manche Attribute in Krankenkassenabrechnungsdaten sehr viele mögliche Merkmalsausprägungen, wie z. B. ICD-Codes (International Statistical Classification of Diseases and Related Health Problems) für Diagnosen [12], ATC-Codes (Anatomisch-therapeutisch-chemische Klassifikation) für Wirkstoffe verordneter Arzneimittel [13] oder OPS-Codes (Operationen- und Prozedurenschlüssel) für durchgeführte Operationen und Prozeduren [14]. In [10] wurden Attribute in einem Datensatz danach klassifiziert, wie unterscheidbar Personen anhand von Attributen bzw. deren Kombination sind. Eine hohe Unterscheidbarkeit kann demnach zu einem höheren Reidentifikationspotenzial beitragen. Durch viele mögliche Merkmalsausprägungen können Datensätze je nach Transformation sehr dünn besetzt werden, was wiederum die Einzigartigkeit bzw. Verknüpfbarkeit von Merkmalskombinationen begünstigt [7].

Exakte Datumsangaben können ebenfalls das Reidentifikationspotenzial erhöhen. Es konnte gezeigt werden, dass in einem Geburtenregister das Geburtsdatum der Mutter und des Kindes ausreichten, um mit einer Wahrscheinlichkeit von 0,88 eine Person reidentifizieren zu können [15]. In Bezug auf Daten zu tödlichen Arzneimittelnebenwirkungen wurde nachgewiesen, dass sich das Risiko einer Reidentifikation deutlich verringert, wenn statt des exakten Sterbedatums nur Monat und Jahr angegeben wurden [16]. Das Risiko erhöhte sich dabei jedoch, wenn die Provinz, in der eine Person lebt, im Datensatz enthalten war, was exakte räumliche Informationen zu einem weiteren Einflussfaktor macht. Dies gilt insbesondere für die Kombination aus zeitlichen und räumlichen Informationen [11].

Nach [17] kann auch die Interpretierbarkeit der Daten zum Reidentifikationspotenzial beitragen, wenn die Daten ohne zusätzliche technische Ressourcen oder Fachwissen interpretiert werden können. Es wird jedoch auch darauf hingewiesen, dass neue Technologien die Interpretierbarkeit von Daten auch für Laien begünstigen können.

### Internationale Standards zum Schutz von Gesundheitsdaten

Als Konsequenz aus der hohen Wahrscheinlichkeit einzigartiger Merkmalskombinationen empfiehlt die European Medicines Agency (EMA) für das öffentliche Teilen von Daten aus klinischen Studien eine Obergrenze für ein akzeptables Reidentifikationsrisiko von 0,09 [18]. Das bedeutet, dass die Wahrscheinlichkeit einer korrekten Reidentifikation von Patientinnen und Patienten höchstens 0,09 betragen darf. Die Obergrenze gilt für die maximale Wahrscheinlichkeit einer Reidentifikation über alle Personen im Datensatz hinweg. Diese Wahrscheinlichkeit berechnet sich üblicherweise aus 1/k, wobei k die Anzahl der Personen im Datensatz mit denselben Merkmalsausprägungen der Schlüsselvariablen ist (k-Anonymität).

International kommt auch der US-amerikanischen Verordnung *Health Insurance Portability and Accountability Act *(HIPAA) eine besondere Bedeutung zu, die die Bereitstellung von medizinischen Einzeldatensätzen regelt [19]. Der Safe-Harbor-Standard listet 18 Attribute, die aus einem Datensatz entfernt bzw. vergröbert werden sollten, bevor dieser geteilt oder veröffentlicht wird. Neben direkt identifizierenden Merkmalen sind hier auch Attribute genannt, die exakte Datumsangaben oder detaillierte Regionalinformationen enthalten. Wenn eines oder mehrere dieser Attribute im Datensatz enthalten sind, gelten die Daten als identifizierbar.

### Verfügbarkeit von externem Wissen

Ob bestimmte Eigenschaften von Gesundheitsdaten ein erhöhtes Reidentifikationsrisiko mit sich bringen, hängt von der Verfügbarkeit von verknüpfbarem externen Wissen ab, das identifizierende Merkmale einer Person, wie z. B. deren Namen, enthält. In [20] wird ein Framework beschrieben, das das Reidentifikationsrisiko als abhängig von Überschneidungen von Merkmalen zwischen 2 Datensätzen, deren einzigartigen Kombinationen sowie den Überschneidungen von Personen zwischen diesen 2 Datensätzen darstellt.

Allgemein verfügbares externes Wissen birgt dabei das größte Risiko. Viele Informationen über einzelne Personen, die in Gesundheitsdaten enthalten sind, sind entweder bereits öffentlich verfügbar, z. B. durch Zeitungsartikel oder Register, oder von der Person selbst öffentlich verfügbar gemacht, z. B. in sozialen Medien. Informationen können jedoch auch nur einem bestimmten Kreis von Personen zugänglich sein. Beispielsweise können bestimmte Merkmalsausprägungen wie Diagnosen für Fachleute beobachtbar sein [11]. Bekannte können demografische Informationen oder Diagnosen kennen. Anderes Zusatzwissen kann recherchierbar sein [11]. Daher sollte bei jeder Risikoanalyse neben den Eigenschaften der Daten berücksichtigt werden, wie wahrscheinlich diese durch Angreifende für eine Reidentifikation verwendet werden können [10].

### Unterschätzung des Reidentifikationspotenzials

Ein Problem bei der Weitergabe sensibler Informationen ist, dass das Reidentifikationspotenzial in der Praxis häufig unterschätzt wird. So gab es in der Vergangenheit wiederholt Fälle, bei denen nicht ausreichend anonymisierte Daten der Allgemeinheit zur Verfügung gestellt wurden. Es konnte beispielsweise gezeigt werden, dass in einem öffentlich zugänglichen Gesundheitsdatensatz, der auf Basis der oben genannten HIPAA-Vorgaben anonymisiert wurde, fast 50 % der Patientinnen und Patienten, die in Unfälle verwickelt waren, reidentifiziert werden konnten, indem ihre Diagnosen und Behandlungsdaten mit Zeitungsberichten über Unfälle verknüpft wurden [21]. Darüber hinaus zeigte beispielsweise [22], dass sich herausfinden lässt, ob Personen mit bestimmten DNS-Profilen in einem Datensatz enthalten sind, selbst wenn nur die Häufigkeiten der Allele veröffentlicht werden. In einem umfangreichen Reidentifikationsexperiment zeigte das U.S. Census Bureau, dass das bisher für den U.S.-Zensus verwendete Zellsperrungsverfahren aufgrund moderner Rechenleistungen und der Vielzahl an Informationen aus anderen Quellen, die für eine Reidentifikation genutzt werden können, in der heutigen Zeit keinen ausreichenden Datenschutz mehr gewährleistet [23]. In einer Übersichtarbeit [24] wurden zudem Reidentifikationsangriffe unter anderem auf Gesundheitsdaten untersucht. Dabei fand sich ein im Allgemeinen hohes Reidentifikationspotenzial, allerdings wurden die gängigen Standards zum Schutz von Daten häufig nicht eingehalten. Dies verdeutlicht die Wichtigkeit besonderer Schutzmaßnahmen, die im Folgenden näher beschrieben werden.

## Möglichkeiten zur Begrenzung des Reidentifikationsrisikos

Ein naheliegender Ansatz, um das Reidentifikationsrisiko zu senken, liegt in der Anonymisierung der Daten. Allerdings bedeutet eine Anonymisierung zwangsläufig auch immer einen Informationsverlust. Um dies zu vermeiden, lassen sich neben der Anonymisierung verschiedene andere organisatorisch-technische Maßnahmen treffen, um das Risiko zu senken. Hier spielt das Konzept der *Five Safes* eine wichtige Rolle.

### Das Konzept der Five Safes

Das Konzept zielt darauf ab, in 5 verschiedenen Dimensionen gewisse Mindestanforderungen zu definieren, die dazu beitragen können, das Risiko zu begrenzen. Die 5 Dimensionen lauten: *Safe Projects* (sichere Projekte), *Safe People* (sichere Personen), *Safe Settings* (sicherer Zugang), *Safe Data* (sichere Daten) und *Safe Outputs* (sichere Ergebnisse). *Safe Projects* setzt voraus, dass es vor der Bereitstellung der Daten einen formalen Prüfprozess gibt. In der Praxis bedeutet dies, dass potenzielle Datennutzende zunächst einen Projektantrag stellen müssen, in dem sie die geplante Nutzung der Daten erläutern. Dieser Antrag wird ethisch und datenschutzrechtlich geprüft, bevor ein Zugriff auf die Daten erfolgen kann. *Safe People* bedeutet, dass der Kreis der Nutzenden reglementiert wird. So beschränken viele Forschungsinstitute den externen Datenzugang auf Wissenschaftlerinnen und Wissenschaftler, die an anderen Forschungseinrichtungen arbeiten. Physikalische und technische Maßnahmen tragen zu *Safe Settings* bei. So können besonders sensible Daten häufig nur vor Ort in den Forschungsdatenzentren der Datenanbieter analysiert werden. Zudem sind in den Forschungsdatenzentren häufig keine eigenen elektronischen Geräte wie Laptops oder Telefone zugelassen. Im Gegensatz zu diesen organisatorisch-technischen Maßnahmen stehen *Safe Data* und *Safe Outputs* im unmittelbaren Zusammenhang mit den bereits angesprochenen Anonymisierungsverfahren. *Safe Data* betrifft die Maßnahmen, die getroffen werden, bevor Externe Zugang zu den Daten erhalten. Im Gegensatz dazu geht es bei *Safe Outputs* darum, die erzielten Analyseergebnisse auf ihr Risiko zu prüfen und vor der Veröffentlichung gegebenenfalls weitere Anonymisierungsmaßnahmen zu treffen, um das Risiko weiter zu verringern. Da die Analyseergebnisse in der Regel der Allgemeinheit zur Verfügung gestellt werden, muss hier eine höhere Anforderung an die Sicherheit gelten, da der Kreis der Nutzenden dieser Ergebnisse nicht mehr auf *Safe People* beschränkt bleibt.

Bei diesem Konzept wird der Unterschied zwischen dem Reidentifikationsrisiko und dem Reidentifikationspotenzial besonders deutlich. Während die letzten beiden Dimensionen nur auf das Potenzial abstellen, liegt der Fokus der ersten 3 Dimensionen darauf, das Risiko zu senken, auch wenn das Potenzial gegebenenfalls hoch bleibt.

### Anonymisierungsansätze

Um *Safe Data* und *Safe Outputs* zu gewährleisten, wird auf eine Vielzahl von Anonymisierungsverfahren zurückgegriffen. Traditionell wird hier zwischen informationsreduzierenden und datenverändernden Verfahren unterschieden. Bei informationsreduzierenden Verfahren wird das Datenschutzrisiko durch Aggregation oder das Entfernen besonders sensibler Merkmale reduziert. Beispielsweise werden Altersangaben nur in 5‑Jahres-Intervallen angegeben oder geografische Angaben von der Kreisebene auf die Bezirksebene aggregiert. Viele Verfahren aus diesem Bereich dienen der Sicherstellung einer mathematischen Definition der Datensicherheit wie der bereits erwähnten k‑Anonymität oder deren Erweiterungen l‑Diversität [25] und *t‑Closeness *[26]. In jüngster Zeit hat in diesem Zusammenhang insbesondere das Konzept der *Differential Privacy* [27] viel Beachtung erfahren, auch deshalb, weil gezeigt werden konnte, dass die bisherigen Definitionen nicht immer einen ausreichenden Datenschutz gewährleisten können [28]. Allerdings fordert das Konzept keine Sicherheitsstandards für die zugrunde liegenden Daten, sondern für die auf Basis der Daten generierten Ergebnisse (*Safe Outputs*).

Im Gegensatz zu den informationsreduzierenden Verfahren bleiben bei datenverändernden Verfahren die detaillierten Informationen erhalten, allerdings werden sie verändert, um das Reidentifikationsrisiko zu senken. Beispiele für diesen Ansatz sind das Aufschlagen eines Störterms beispielsweise bei Einkommensangaben oder das zusätzliche Vertauschen einzelner Merkmalsausprägungen (*Swapping*). Allerdings wurde in den letzten Jahren in mehreren simulierten und echten Datenangriffen gezeigt [2, 3, 29], dass die traditionell eingesetzten Verfahren in Zeiten leistungsfähiger Rechner und ubiquitär verfügbarer Daten nicht mehr ausreichen, die Daten ausreichend zu schützen. Um einen ausreichenden Schutz auch bei einer allgemeinen Bereitstellung, wie sie in verschiedenen Initiativen zu offenen Daten gefordert wird, sicherzustellen, müssten diese Verfahren in so einem starken Umfang eingesetzt werden, dass die resultierenden Daten für die meisten Analysezwecke nutzlos würden.

Eine Möglichkeit, diese Herausforderung insbesondere bei sensiblen Daten zu adressieren, stellt die Bereitstellung synthetischer Daten dar. Bei diesem Verfahren, das erstmals in [30] vorgeschlagen wurde, werden statt der Originaldaten künstlich erzeugte Daten zur Verfügung gestellt, die in ihren Verteilungseigenschaften den Originaldaten entsprechen. Erreicht wird dies, indem komplexe Modelle an die Originaldaten angepasst werden. Die synthetischen Daten werden dann erzeugt, indem Zufallszüge aus den angepassten Modellen gezogen werden. Wurden in den Anfängen überwiegend parametrische Modelle, wie beispielsweise lineare Regressionsmodelle zur Erzeugung synthetischer Daten, verwendet (zum Beispiel in [31] oder [32]), kommen in den letzten Jahren zunehmend Verfahren des maschinellen Lernens zum Einsatz [33–35]. Eine tiefergehende Einführung in das Thema bieten beispielsweise [36] und [37].

Synthetische Daten werden in den letzten Jahren zunehmend in der Praxis eingesetzt [32, 38–42]. Da die Datensynthetisierung durch ihren hohen Grad der Anonymisierung insbesondere für Datensätze geeignet ist, die sensible Informationen erhalten, ist es wenig überraschend, dass das Verfahren zunehmend auch im Gesundheitssektor auf großes Interesse stößt. So werden beispielsweise synthetische Daten an der US-amerikanischen *Oregon Health and Science University* eingesetzt, um Studierenden die Herausforderungen bei der Analyse von klinischen Daten zu vermitteln [43]. Die *Centers for Medicare and Medicaid* (CMS) in den USA bieten synthetische *Medicare Claims Public Use Files* (SynPUFs) an, die zur Entwicklung von Analysecode verwendet werden können [44]. In den USA wird zudem durch die Non-Profit-Organisation *MITRE* eine Open-Source-Software zur Erzeugung synthetischer Gesundheitsdaten angeboten [45]. Die Organisation bietet auch einen Zugang zu synthetischen Daten für den Bundesstaat Massachusetts, die über eine API^4^ ausgewertet werden können [46]. Die *United States National COVID Cohort Collaborative* (N3C) haben eine synthetische Version ihrer gesammelten elektronischen Krankenakten erstellt, um einen breiteren Zugang zu diesen Daten zu ermöglichen. In einer umfangreichen Evaluationsstudie kommen sie zu dem Ergebnis, dass die synthetischen Daten für eine Vielzahl von Auswertungen nützliche Ergebnisse liefern [47]. Auch das *National Center of Health Statistics* (NCHS) arbeitet mit synthetischen Daten: In den *Public-Use Linked Mortality Files*, die im Internet frei zugänglich verfügbar sind, wurden einzelne Variablen, wie zum Beispiel die Todesursache, durch synthetische Versionen ersetzt [48].

## Fazit und Ausblick

Zusammenfassend können viele Faktoren zu einem hohen Reidentifikationspotenzial beitragen. Dies können Eigenschaften der Daten sein, wie die Einzigartigkeit von Merkmalsausprägungen und deren Kombinationen oder zeitliche und räumliche Informationen. Die Wahrscheinlichkeit, ob eine Reidentifikation tatsächlich erfolgen kann, hängt jedoch von öffentlich oder nicht-öffentlich verfügbarem externen Wissen sowie von weiteren Faktoren ab. Das Reidentifikationspotenzial darf nicht unterschätzt werden und zur Mitigation sollten in verschiedenen Bereichen technisch-organisatorische Maßnahmen entsprechend dem Konzept der *Five Safes* eingesetzt werden. Hierbei stehen unter anderem klassische Anonymisierungstechniken und die Generierung synthetischer Daten zur Verfügung.

Bei den traditionellen Verfahren der Anonymisierung gibt es gerade bei sensitiven Daten wie Gesundheitsdaten das Problem, dass sowohl die informationsreduzierenden als auch die datenverändernden Verfahren derart umfänglich angewandt werden müssten, um einen ausreichenden Schutz zu gewährleisten, dass die resultierenden Daten für Forschungszwecke praktisch nutzlos würden [49]. Bei synthetischen Daten stellt insbesondere die Akzeptanz eine große Herausforderung dar [50]. Woher sollen die Forschenden die Gewissheit nehmen, dass die Ergebnisse, die sie auf Basis der synthetischen Daten erhalten, hinreichend nahe an den Ergebnissen auf Basis der Originaldaten liegen?

Daher wird in der Praxis oft ein Mittelweg gewählt, bei dem die Forschenden auf synthetischen Daten ihre Analyseprogramme schreiben und diese anschließend über eine kontrollierte Datenfernverarbeitung auf den Echtdaten ausführen oder auf Echtdaten basierende Zwischenergebnisse ausgegeben werden. Auch für das im Aufbau befindliche Forschungsdatenzentrum Gesundheit (FDZ Gesundheit) am Bundesinstitut für Arzneimittel und Medizinprodukte (BfArM) wird ein solcher Ansatz verfolgt, wobei statt synthetischer Daten auch klassisch anonymisierte Daten innerhalb einer geschützten virtuellen Analyseumgebung bereitgestellt werden können. Ein Vergleich dieser beiden Ansätze wird im Rahmen eines aktuell laufenden Forschungsprojekts durchgeführt, das im vorliegenden Heft vorgestellt wird.

## Footnotes

1 https://www.rnd.de/politik/corona-zahlen-aerztepraesident-reinhardt-kritisiert-mangelnde-datenlage-vorbild-grossbritannien-3SYNUVD4RVFAXLJWWKN7DUZNSQ.html (Zugegriffen: 10. Juli 2023).

2 https://www.nature.com/articles/d41586-022-04422-9 (Zugegriffen: 10. Juli 2023).

3 https://de.statista.com/statistik/daten/studie/155823/umfrage/gkv-pkv-mitglieder-und-versichertenzahl-im-vergleich/ (Zugegriffen: 10. Juli 2023).

4 Eine API (Application Programming Interface) ist ein Satz von Befehlen, Funktionen, Protokollen und Objekten, um eine Software zu erstellen oder mit einem externen System zu interagieren.
